# Neurocysticercosis With Internal Carotid Artery and Middle Cerebral Artery Vasculitis and Stenosis

**DOI:** 10.7759/cureus.27407

**Published:** 2022-07-28

**Authors:** Pradeep Kumar, Arun Prasad, Subhash Kumar

**Affiliations:** 1 Pediatrics, All India Institute of Medical Sciences, Patna, IND; 2 Radiodiagnosis, All India Institute of Medical Sciences, Patna, IND

**Keywords:** infarction, facial palsy, stroke, hemiparesis, ncc

## Abstract

A seven-year-old female child presented with sub-acute onset headache, vomiting, and aphasia with right-sided upper motor neuron (UMN) type hemiparesis and ipsilateral UMN type facial nerve weakness. Her coagulation profile and thrombotic profile were normal. MRI brain with magnetic resonance angiography (MRA) detected neurocysticercosis causing secondary vasculitis and narrowing of supraclinoid left internal carotid artery (ICA) and middle cerebral artery (MCA). The patient was given aspirin along with steroids and albendazole. She improved gradually, and her hemiparesis and facial nerve palsy improved completely by three months and aphasia by four months.

## Introduction

Neurocysticercosis is one of the most common causes of neurological manifestation by parasitic infection worldwide. The highest prevalence of cysticercosis is in communities where there is close contact between humans and pigs, and where hygiene standards are low. It is a disease produced by poor sanitation, lack of a proper water supply and sewage system, and poor personal hygiene [1].

The clinical presentations are pleomorphic depending on the stage and location of cysts in the nervous system. Most children (>80%) present with seizures, particularly partial seizures; headache and vomiting are seen in about a third of cases. Solitary enhancing lesions are the most common finding in neuroimaging [2]. Focal neurological deficit is seen in 4% of cases in children [3]. Cerebrovascular complications of cysticercosis have often been reported, most of these relate to small vessel angitis, clinically manifesting as lacunar infarctions. Cerebral infarction due to major intracranial vessel involvement is rare [4-5].

We present a case of neurocysticercosis with conglomerated cystic lesions and irregular thick meningeal enhancement in the Sylvian fissure with occlusion of the left internal carotid artery (ICA) terminus and middle cerebral artery (MCA) with infarction in the left MCA territory shown by MRI brain with magnetic resonance angiography (MRA).

## Case presentation

A seven-year-old female child presented with headache and vomiting for 16 days and right-sided upper and lower limb weakness for four days. The child was apparently well 16 days back then she developed a diffuse headache and was associated with non-projectile vomiting two to three episodes per day. This was followed by weakness in the right-side upper and lower limbs, which gradually led to difficulty in walking. There was no history of fever, trauma, recent immunization, or seizure. There was no history of upper respiratory infection or earache in the recent past. Her birth history and developmental history were insignificant.

On general physical examination, her vitals were normal. On central nervous system examination, she was conscious, cooperative, and oriented. Bilateral pupil size was equal, with normal reaction to light and there was no papilledema. She had increased tone and 4/5 power in the right upper and lower limbs. She also had right-sided upper motor neuron (UMN) type facial palsy and aphasia with increased knee and ankle jerks on the same side. Planter reflex on the right side was upgoing. She had normal bladder and bowel functions, no sensory involvement, and no signs of meningeal irritation. The rest of the systemic examinations were normal.

A provisional diagnosis of subacute stroke complicating right-sided UMN type hemiparesis with ipsilateral UMN type seventh cranial nerve palsy was made and baseline investigations and coagulation profile with MRI brain with angiography were planned.

Complete blood count was otherwise normal with increased eosinophil counts. Prothrombin time was slightly raised with the international normalized ratio (INR) within the range. Erythrocyte sedimentation rate (ESR) and C-reactive protein (CRP) were normal. Her electrolytes, calcium, phosphate, liver function tests with protein, and renal function tests were grossly within the normal range. X-ray chest was normal, Manteaux test was negative, Ziehl-Neelsen (ZN) stain of sputum for acid-fast bacilli was negative, and cartridge based nucleic acid amplification test (CBNAAT) did not detect mycobacterium tuberculosis in sputum. Her thrombotic profile showed Protein S, Protein C, anti-thrombin III and anti-phospholipid immunoglobulin M (IgM) 0.22 optical density (OD) ratio (<0.8) were within the normal limit for the reference range. Lupus anti-coagulant was absent too (Table 1).

**Table 1:** Investigation reports. ESR, erythrocyte sedimentation rate; IgM, immunoglobulin M; OD, optical density; CBNAAT, cartridge based nucleic acid amplification test; USG, ultrasonography; ICA, internal carotid artery; MCA, middle cerebral artery

Investigation	Result	Normal value
Hemoglobin (g/dL)	12.5	11.5–14.5
Platelets (per cmm)	290 × 103	150–450 × 103
Leucocyte count (per cmm)	12,500	4,000–11,000
Differential leucocyte count (%):		
Neutrophils	43.0	40-80
Lymphocytes	40.6	20-40
Monocytes	1.2	2-10
Eosinophils	13.2	1-6
Basophils	2.9	0-1
Prothrombin time (s)	16	11-14
ESR (mm in the first hour)	08	0-10
Serum bilirubin (total) (mg/dL)	0.74	0.3–1.2
Serum bilirubin (direct) (mg/dL)	0.44	<0.3
Aspartate aminotransferase (IU/L)	20	<31
Alanine aminotransferase (IU/L)	17.2	10–28
Alkaline phosphatase (IU/L)	245.9	100–290
Total protein (g/dL)	6.01	6.4-8.1
Albumin (g/dL)	3.73	3.5-5.6
Globulin (g/dL)	2.29	2.0-3.5
Blood urea (mg/dL)	29.4	13–43
Serum creatinine (mg/dL)	0.42	0.7–1.3
Serum sodium (mmol/L)	137.9	135–145
Serum potassium (mmol/L)	4.0	3.5–5
Serum calcium (mg/dL)	10.15	8.8–10.8
Serum phosphate (mg/dL)	4.86	3.2–5.8
CRP (mg/L)	2.8	0.8–7.9
Protein C (%)	104	65–140
Protein S (%)	76	70–140
Antithrombin-III (%)	105.8	80–120
Anti-phospholipid IgM (OD ratio)	0.22	<0.8
Lupus anticoagulant	Absent	
Tuberculosis work-up (Mantoux, chest-X ray, gastric aspirate for acid-fast bacilli and CBNAAT)	Negative	
USG-B scan eye	No cysticerci	
MRI brain with angiography	Conglomerated cystic lesions and irregular thick meningeal enhancement in the Sylvian fissure with occlusion of the left ICA terminus and MCA with subacute infarcts in the left MCA territory	

**Figure 1:**
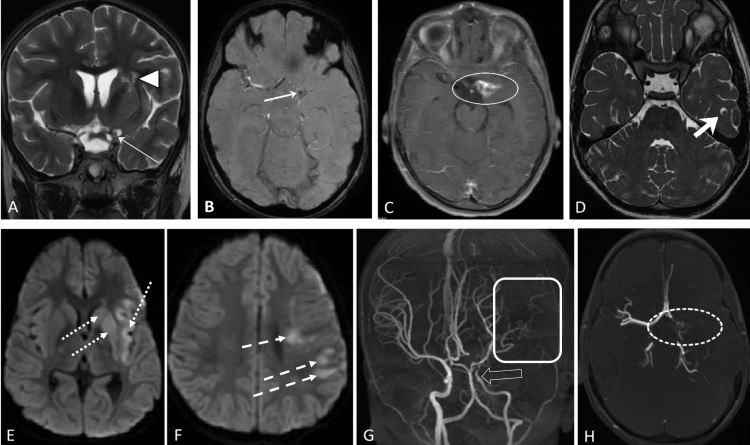
MRI and MRA brain. MRI brain, plain and contrast imaging A) Coronal T2 weighted image showing conglomerated hyperintense cystic lesions with an eccentric hypointense nodule in the left Sylvian fissure (thin white arrow), in the region of the ICA bifurcation, irregular T2 hyper intensity is seen in left putamen, caudate and internal capsule (arrowhead). B) Axial SWAN image shows a tiny hypointensity (thick white arrow) representing calcified mural nodule of the cysticercal cyst. C) Post contrast axial T1 fat-suppressed image showing irregular thick enhancement (solid oval). D) Axial FIESTA-C image showing a cysticercal cyst with eccentric nodule in the left temporal lobe (thick short arrow). E) Axial diffusion weighted image at the level of the basal ganglia showing restricted diffusion lesion, representing infarcts in left MCA territory in the left insula, putamen, and caudate nuclei (dotted white arrows). F) Axial diffusion weighted image at the level of centrum semiovale showing restricted diffusion lesion, representing infarcts in left MCA territory in the left frontal and parietal lobes (dashed arrows). G) MRA, MIP image, showing nonvisualization of left MCA, narrowing of the left supraclinoid ICA (hollow white arrow) and paucity of vessels in the left cerebral hemisphere (white rectangle) compared to the right side. H) Axial thick MIP image showing tiny irregular vessels, possibly representing proliferating collaterals (dashed oval). ICA, internal carotid artery; SWAN, star-weighted angiography; FIESTA, fast imaging employing steady-state acquisition; MCA, middle cerebral artery; MRA, magnetic resonance angiography; MIP, maximum intensity projection; ICA, internal carotid artery

An MRI scan of the brain with angiography was done which showed conglomerated cystic lesions and irregular thick meningeal enhancement in the Sylvian fissure with occlusion of the left ICA terminus and MCA with subacute infarcts in the left MCA territory (Figure 1).

The ultrasonography (USG)-B scan showed no cysticerci in the eyes. She was treated with oral aspirin along with prednisolone for two weeks. Oral albendazole 15 mg/kg/day was started from day two of the steroid for 28 days in divided dosage. She is under our regular follow-up, her hemiparesis and facial paralysis had gradual improvement over three months, and she could walk independently now. A follow-up MRI brain with angiography has been planned to see radiological improvement.

## Discussion

Cerebrovascular events are a common complication of pediatric neurocysticercosis, but most of them are less frequently recognized. Various mechanisms have been described by which cerebral vessels may be involved [4-6]. Alarcón et al. in their study of 420 adult patients concluded that occlusion of the small cortical or penetrating vessels at the base of the brain caused by arteriopathy was the most common mechanism of stroke in neurocysticercosis. However, they did not find any occlusion of large vessels despite the CT brain showing cortical infarction [4]. Cantú et al. reported a series of nine patients (mean age 32 years) with neurocysticercosis and stroke found occlusion or stenosis of the MCA, ACA, posterior cerebral artery (PCA), or basilar artery but not ICA either by cerebral angiography or trans-cranial doppler [5]. Barinagarrementeria et al. in their study of 28 patients with a subarachnoid form of neurocysticercosis, found that 53% had angiographic evidence of middle or PCA occlusion and the most commonly involved vessels were the MCA and the PCA [7]. Jha and Kumar in their series of six children presenting with stroke and neurocysticercosis revealed that the most common involved vessels were the MCA and the rest were the ACA and PCA but no ICA [8]. Vieira et al. reported a 42-year-old lady, who presented with severe headache and vomiting. They could not do an MRI brain. Further investigating this case by digital subtraction angiography (DSA), showed an aneurysmal dilatation on the frontal M2 segment of the left MCA associated with multiple areas of irregular caliber corresponding to arterial spasm. After performing a wide microsurgical dissection of the Sylvian fissure, they revealed multiple cysts in the left carotid and Sylvian cisterns associated with a dense inflammatory exudate involving the MCA. Later, histopathological examination was suggestive of neurocysticercosis [9]. Opara reported cortical blindness in a 14-year-old-boy, who had neurocysticercosis. The author hypothesized the cause to be likely PCA occlusion (Table 2) [10].

**Table 2:** Involvement of intracranial vessels in neurocysticercosis. MCA, middle cerebral artery; ACA, anterior cerebral artery; PCA, posterior cerebral artery; ICA, internal carotid artery; SAH, subarachnoid hemorrhage *Mean age was not found in the article.

Author (year of publication)	Presentation	Number of patients	Age (years)	Observation
Alarcón et al. (1992) [4]	Stroke	420	Mean 52 (17-86)	Occlusion of the small cortical or penetrating vessels at the base of the brain, sparing large vessels of brain
Cantú et al. (1998) [5]	Stroke	9	Mean 32 (16-44)	Occlusion or stenosis of the MCA, ACA, PCA, or basilar artery sparing ICA
Barinagarrementeria and Cantú (1998) [7]	Stroke	28	Mean 37 (16-58)	53% had angiographic evidence of MCA or PCA occlusion
Jha and Kumar (2000) [8]	Stroke	6	7-30*	Involvement of MCA, ACA, and PCA sparing ICA
Vieira et al. (2019) [9]	Severe headache and vomiting due to SAH	1	42	Aneurysmal dilatation of left MCA
Opara (2022) [10]	Cortical blindness	1	14	Likely PCA occlusion

In our patient, it was neurocysticercosis with conglomerated cystic lesions and irregular thick meningeal enhancement in the Sylvian fissure with occlusion of the left ICA terminus and MCA with subacute infarcts in the left MCA territory. One of the accepted mechanisms of inflammation of arteries is that degenerating parasite-induced local arachnoiditis in the host in adjacent areas, then invades vessel walls by inflammatory cells, resulting in an occlusive endarteritis characterized by thickening of the adventitia, fibrosis of the media, and endothelial hyperplasia [11].

Segmental narrowing and occlusion of small- and medium-sized arteries arising from the circle of Willis and its major branches may be because of inflammatory reactions surrounding subarachnoid cysticerci [12].

## Conclusions

The MCA, anterior cerebral artery, and PCA are commonly involved vessels in pediatric and adult neurocysticercosis with cerebrovascular events. ICA occlusion and stenosis is an extremely rare presentation of pediatric neurocysticercosis. MRI brain with angiography is a must to detect these lesions in patients who present with hemiparesis or any other focal neurological deficit.
